# Relevance research between the expression of p16^INK4a^, Notch1, and hTERC genes: The development of HPV16‐positive cervical cancer

**DOI:** 10.1002/jcla.23207

**Published:** 2020-01-24

**Authors:** Wenyan Huo, Shuaiyu Zhai, Yanbo Wang, Xin Qiang, Risu Na, Hua Gui, Ningjin Wu, Yaning Cao, Haihua Bai

**Affiliations:** ^1^ Affiliated Hospital of Inner Mongolia University for the Nationalities Tongliao China; ^2^ Inner Mongolia Engineering Research Center of Personalized Medicine Tongliao China; ^3^ College of life sciences Inner Mongolia University for Nationalities Tongliao China; ^4^ Medical school Inner Mongolia University for Nationalities Tongliao China; ^5^ XiangYa school of Medicine Central South University Changsha China; ^6^ School of Life Science Inner Mongolia University Huhehaote China

**Keywords:** cervical cancer, HPV16, hTERC gene, Notch1 gene, p16^INK4a^ gene, SiHa cells

## Abstract

**Background:**

GLOBOCAN 2018 latest data show cervical cancer ranks fourth in morbidity and mortality among women. Many genes in cervical lesions differ in sensitivity and specificity. However, the diagnostic molecules for early cervical cancer are not very clear. This paper screens biomarkers for early molecular diagnosis of Mongolian patients with cervical cancer.

**Methods:**

Immunohistochemical SP method was used to detect the expression of p16^INK4a^ and Notch1 protein in paraffin sections of 226 Mongolian patients with HPV16‐positive cervical lesions after pathological examination, and 100 of them were randomly selected by fluorescence in situ hybridization to detect hTERC gene. The HPV16‐binding human cervical cancer SiHa cell line was used to silence the expression of HPV16 E6/E7 gene by RNA interference, and the expression of p16^INK4a^, Notch1, and hTERC genes and protein expression levels were detected by RT‐PCR and Western blot.

**Results:**

The positive expression rates of p16^INK4a^, Notch1, and hTERC genes in HPV16‐positive cervical cancer, CIN‐III, CIN‐II, CIN‐I, uterine leiomyoma, and chronic cervicitis were significantly different (*P* < .05); the positive expression rates of the three genes were also significantly different in the same type of cervical lesions (*P* < .05); RNA interference can effectively inhibit HPV16 E6/E7, p16^INK4a^ and Notch1 gene expression, but has no effect on hTERC gene expression.

**Conclusion:**

The p16^INK4a^ gene can be used as a biomarker for early screening of cervical cancer, and the hTERC gene can be used to confirm the clinical diagnosis of cervical cancer.

## 1 INTRODUCTION

In the past 50 years, due to the extensive development of gynecological census, the incidence and mortality of cervical cancer have decreased significantly. However, it is still the third most common malignant tumor after breast and colorectal cancer and is one of the important causes of female death worldwide.^1^ Cervical cancer (CC) is the most common malignant tumor of the female reproductive system, which seriously endangers women's life and health. Cervical cancer and precancerous lesions are currently considered to be a persistent, progressive, multifactorial, and multi‐step disease, and human papilloma virus (HPV) infection is the leading cause of cervical cancer development.^2^ The positive rate of HPV infection in patients with cervical cancer is as high as 99%.^3^, ^4^, ^5^

Human papilloma virus is a non‐enveloped double‐stranded circular DNA virus consisting of 7900 base pairs. The HPV gene structure basically includes three important regions: early region (E), late region (L), and long control region (LCR). The early region (E) encodes products E6 and E7, and their abnormal expression is a key event in the malignant development of infected cells, which is related to various alteration pathways of viruses and cells.^6^ Clinically, HPV is classified into the low‐risk type and high‐risk type according to the virulence of HPV subtype or the risk of cancer. Among them, high‐risk HPV infection has a closer relationship with the development of cervical cancer and its precancerous lesions. There are two states after HPV infection of the cervix, which are free and combined, and the persistent infection of HPV in the combined form is an important cause of cervical cancer development.^7^ Wang et al^8^ found that especially high‐risk HPV16/18 is closely related to the occurrence of cervical cancer. HPV16 is recognized as the most important genotype for the development of squamous cell carcinoma and adenocarcinoma worldwide.^9^ Therefore, this study used HPV16‐infected cervical lesions and cervical cancer cells as the research object. By detecting the differential expression of p16^INK4a^, Notch1, and hTERC genes, the relationship between these genes and the occurrence and development of Mongolian patients with cervical cancer was analyzed, and screen the best reference biomarkers for early diagnosis of cervical cancer in Mongolian population, and establish the relationship between these three genes and HPV16 infection.

The P16^INK4a^ gene is a tumor suppressor gene directly involved in the negative feedback regulation of the cell cycle. Inactivation of the P16^INK4a^ gene can lead to excessive cell proliferation, and cells that are not fully developed in the G1 phase enter the S phase in advance, resulting in tumorigenesis. Studies on cervical cancer tissue have found that P16^INK4a^ gene deletion or mutation is rare. In contrast, p16^INK4a^ is overexpressed in cervical cancer and precancerous lesions caused by 100% HR‐HPV infection. However, it is not expressed in HPV‐negative cervical cancer and normal tissues.^10^ Therefore, p16^INK4a^ gene expression is important for precancerous screening.

The Notch1 signaling pathway plays an important role in some key steps regulating cell differentiation, proliferation, and apoptosis. Notchl expression is increased in cervical intraepithelial neoplasia (CIN) and cervical cancer tissues. Laura et al believe that the carcinogenesis of the normal cervical epithelium may be related to the increased expression of Notchl protein, leading to the development of cervical cancer.^11^ The hTERC gene has a certain inhibitory effect on apoptosis and is closely related to tumorigenesis.^12^ In recent years, the National Institutes of Health research on cervical cancer showed that the majority of cervical epithelial cell carcinogenesis is accompanied by an increase in 3q copy number. The human telomerase RNA component (hTERC gene, located at 3q26.3) may be the most important gene involved. Meng‐Lan O et al^13^ found that the hTERC gene is activated in the early stages of cervical cancer. Therefore, it is possible to diagnose cervical cancer based on the activity of telomerase and predict the development of cervical cancer. In summary, p16^INK4a^, Notch1, and hTERC genes are abnormally expressed in HPV‐infected cervical diseases.^14^, ^15^, ^16^ However, there are large differences in the reports of p16^INK4a^‐positive expression rates in cervical tissues in different articles.^17^ We speculate that the expression levels of these three genes are possibly to be different in population expression. Studies have shown that the E6 and E7 proteins produced by HPV16 E6 E7 mRNA can inactivate cell growth inhibitory genes such as p53 and pRB.^18^ The increased risk of cervical cancer is due to the overexpression of E6 and E7 oncoproteins. In this study, Mongolian cervical cancer patients was designed to detect gene expression of p16^INK4a^, Notch1, and hTERC in HPV16‐infected tissues including cervical cancer, CIN‐III, CIN‐II, CIN‐I, uterine leiomyoma, and chronic cervicitis. The human cervical cancer SiHa cell line was used as the research object, and the HPV16 E6/E7 gene expression was silenced by RNA interference. The expression of p16^INK4a^, Notch1, hTERC, and HPV16 E6/E7 genes and proteins were detected by RT‐PCR and Western blot. The relationship between the three genes and HPV16 E6/E7 gene was analyzed. In‐depth study of the three genes p16^INK4a^, Notch1 and hTERC and their carcinogenesis will help to guide the prevention, early diagnosis, treatment and prognosis of cervical lesions.

## 2 MATERIALS AND METHODS

### 2.1 Paraffin sections of cervical pathological tissue and positive HPV16 were determined

Pathological tissue was collected at the Affiliated Hospital of Inner Mongolia University for Nationalities from September 2016 to September 2017. The Mongolian cervical patients identified by 23 kinds of HPV genotyping detection kit (PCR fluorescence probe method) were provided by Guangzhou Caipu Biotechnology Co., Ltd. We strictly followed the instruction manual for extracting HPV DNA and PCR amplification and used the ABI Quantstudio DX fluorescence quantitative PCR (ABI company) to analyze HPV DNA genotyping amplification detection. Among the 226 HPV16‐positive patients, 45 were cervical cancer, 35 were CIN‐III, 32 were CIN‐II, 38 were CIN‐I, 42 were uterine fibroids, and 34 were chronic cervicitis. The average age was 43.7 years, ranging from 20 to 71 years old (Table S1). All patients were not treated with physical therapy, biological therapy, drug therapy, chemotherapy, radiotherapy, or surgery. And all the sections were diagnosed with the same opinion obtained by two senior professors of pathology.

### 2.2 Immunohistochemistry

Immunohistochemistry was performed using SP‐9000 DAB staining solution (streptavidin‐biotin method) kit manufactured by Beijing Zhongshan Golden Bridge Biotechnology Co., Ltd. Abcam mouse anti‐human p16 monoclonal antibody and rabbit monoclonal antibody Notch1 were used. The paraffin‐embedded tissue sections with a thickness of 4 μm were used as specimens. Immunohistochemical examination was performed on 226 patients with HPV16‐positive tissue, and brown‐yellow coloring was recorded as positive expression. Greenspan semi‐quantitative method^19^: 0 is that the cells are basically not colored, 1 is a lighter coloration, 2 is a moderate coloring, and 3 is a deeper coloring. The percentage of cells stained for all counted cells is the positive cell rate: Generally, 0 points are recorded as 0 points, 11 points are recorded as 1 point, and 51%‐75% is counted as 2 points. It is recorded as 3 points at around 76%. The positive coefficient is the number of the staining degree score of each slice multiplied by the percentage of the stained cells. The positive coefficient is 0 for the negative (−), and the positive coefficient is 1‐2. Expressed as weakly positive (+), positive coefficient is 3 to 4 points is positive (++), positive coefficient is 5‐9 points is expressed as strongly positive (+++), and overexpression is (++‐+++).

### 2.3 Fluorescence in situ hybridization (FISH)

100 samples of 4 μm thick paraffin sections were selected from 226 cases of HPV16‐positive tissues (25 cases of cervical cancer, 10 cases of CIN‐III, 10 cases of CIN‐II, 10 cases of CIN‐I, 15 cases of uterine leiomyoma, and 30 cases of chronic cervicitis) and were tested by FISH using the probe GLP TERC/CSP3 and corresponding kits purchased from Guangzhou Ambition (LBP) Pharmaceutical Technology Co., Ltd. (batch number: 201704001). The Olympus BX53 fluorescence microscope was used to observe the interphase fluorescence hybridization signal under the excitation of DAPI/Green/Orange trichrome filter. Image analysis using FISH analysis software provided by Bei Ang Medical Co., Ltd. identifies cells with an intact nucleus for detection and establishes thresholds for chronic cervicitis tissue. Each sample has a random count of more than one hundred cells, and if the detected value exceeds the threshold, the result is considered positive; if the detected value is less than the threshold, the result of the determination is negative; and if both of them are equal, the count should be increased. According to the FISH kit, normal cells: there are two red and green signals in a single‐cell nucleus; Abnormal cells: The red signal in the single‐cell nucleus is ≥3, the hybridization of the test gene of the whole slide and the hTERC/CSP3 two‐color probe is comprehensively evaluated.

### 2.4 Cell culture and HPV16 E6/E7 siRNA transfection of SiHa cells

The SiHa cell line was purchased from Procell and cultured in MEM medium containing 10% fetal bovine serum. HP V16 E6/E7 siRNA designed and synthesized by Guangzhou Ruibo Company, Sense(5′‐3′): GCACACACGUAGACAUUCGdTdT; Antisense(5′‐3′): CGAAUGUCUACGUGUGUddTdT and negative control SiRNA: siN05815122147 NControl_05815. Purchase GIBCO's Lipofectamine 2000 and construct the transfection complex according to the instructions for transfection reagents. SiHa cells in a 6‐well culture plate were transfected with a non‐interference group (nontransfection), a nonsense interference control group (siNControl), and a siRNA transfection group (siHPV16 E6/E7).

### 2.5 RT‐PCR and Western blot

Total RNA from the above three groups of SiHa cells was prepared using TRIzol (Invitrogen), and OD values of 260 nm and 280 nm wavelength were measured by NanoDrop2000 ultra‐micro spectrophotometer, and Abs260/Abs280 ratio and RNA concentration were recorded. cDNA was synthesized using a universal RT‐PCR kit purchased from Beijing Dingguo Changsheng Biotechnology co., LTD. 3ug of total RNA was taken as the template, and cDNA was amplified using gene‐specific primers (Table 1). The amplified products were separated by 2% agarose gel electrophoresis.

**Table 1:** Gene‐specific primers for the RT‐PCR

Gene	Sense (5′ → 3′)	Antisense (5′ → 3′)
GAPDH	TTTGGTATCGTGGAAGGAC	AAAGGTGGAGGAGTGGGT
HPV16 E6/E7	ATGCATGGAGATACACCT	TTATGGTTTCTGAGAACA
Notch1	GAGAAGGGAAGTTGAACGAGC	CACATGGCAACATCTAACCC
P16^INK4a^	AGCATGGAGCCTTCGG	CAATCGGGGATGTCTGA
hTERC	CTGGGAGGGGTGGTGGCCATTT	CGAACGGGCCAGCAGCTGACAT

Total protein was extracted from three groups of SiHa cells by using the total cell protein extraction kit (Solarbio), and the concentration was measured on a Nanodrop 2000 ultra‐micro spectrophotometer (Gene Co., Ltd.). Take 0.5 mg of total protein and transfer to PVDF membrane (Millipore). Specific protein detection using Western blot kits and antibodies purchased from Proteintech, the PVDF membrane was placed in a Bio‐Rad scanner for photography.

### 2.6 Statistical analyses

Statistical analysis was performed using SPSS 21.0 statistical software. The chi‐square test was performed. The difference was statistically significant at *P* < .05. The correlation test was performed using the Spearman rank correlation analysis.

## 3 RESULTS

### 3.1 The gene expression of p16^INK4a^, Notch1, and hTERC was significantly different in HPV16‐positive cervical lesions at different stages

**Figure 1:**
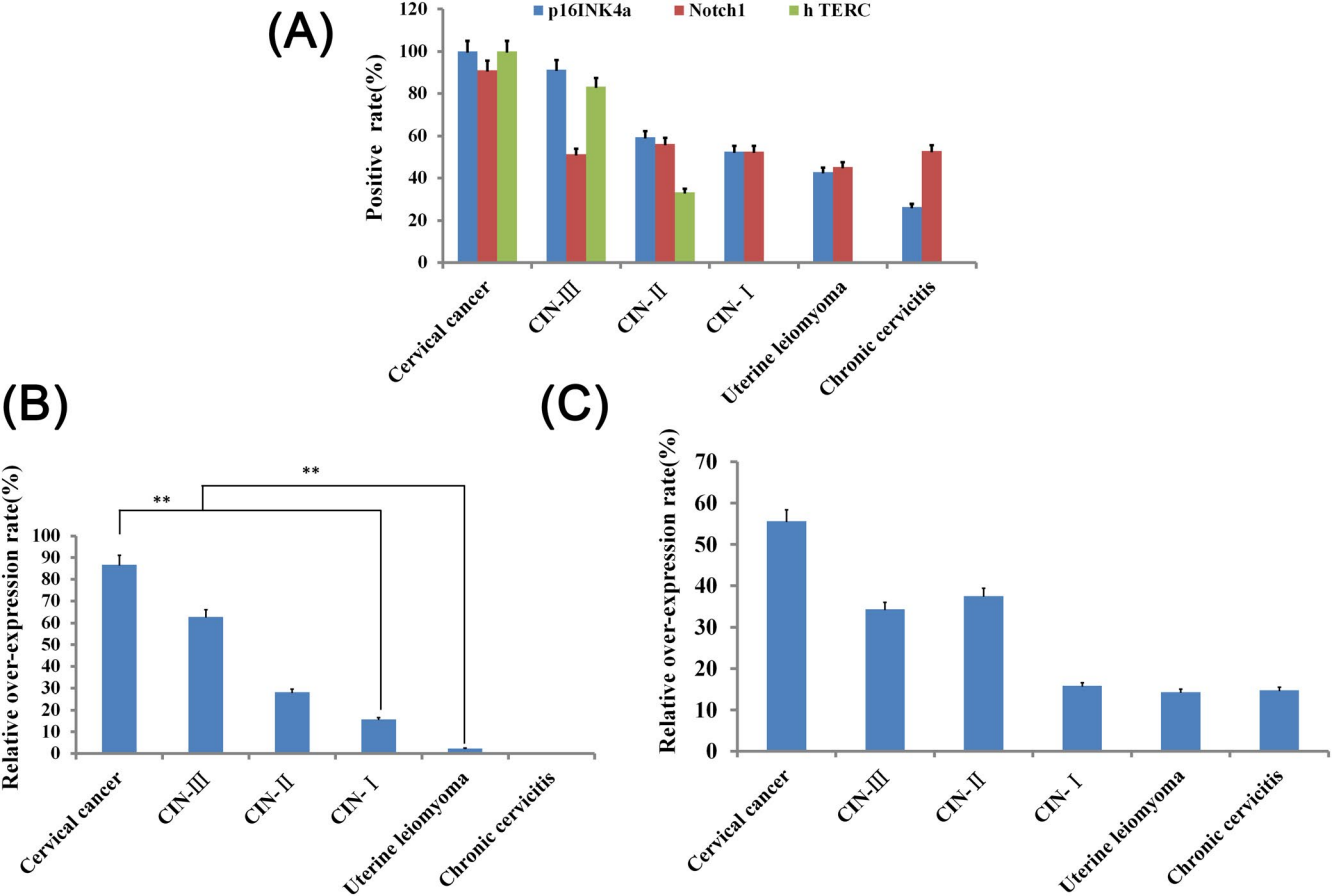
The expression of three genes in HPV16 positive cervical lesions. (A) The expression of p16^INK4a^, Notch 1 and hTERC genes in the same type of cervical lesions; (B) overexpression of p16^INK4a^ in cervical lesions; (C) overexpression of Notch 1 protein in cervical lesions

The detection of HPV16‐positive cervical lesions by immunohistochemistry and fluorescence in situ hybridization showed that the positive expression rates of p16^INK4a^ were 100.00 (45/45), 91.43 (32/35), 59.38 (19/32), 52.63 (20/38), 42.86 (18/42), and 26.47 (9/34); the positive expression rates of Notch1 were 91.11 (41/45), 51.43 (18/35), 56.25 (18/32), 52.63 (20/38), 45.23 (19/42), and 52.94 (18/34); the positive amplification rate of hTERC was 100.00 (25/25), 83.33 (10/12), 33.33 (4/12), 0 (0/12), 0 (0/9), and 0 (0/30) in cervical cancer, CIN‐III, CIN‐II, CIN‐I, uterine leiomyoma, and chronic cervicitis, respectively; and all differences were significant (Table 2). The p16^INK4a^ was the most significant. What's more, the positive expression rate of the three types of HPV16‐positive cervical lesions was also significantly different (*χ* ^2^ = 149.414, *P* = .000) (Figure 1A). The p16^INK4a^ and Notch1 are overexpressed in cervical lesions, especially obvious in cervical cancer and CIN‐III, while the p16^INK4a^ gene performs more prominently (Figure 1B,C).

**Table 2:** Expression of p16^INK4a^, Notch1, and hTERC in cervical lesions

	Tissue types	Cervical cancer	CIN‐III	CIN‐II	CIN‐I	Uterine leiomyoma	Chronic cervicitis	*χ* ^2^	*P*
P16^INK4a^	Cases, n	45	35	32	38	42	34	151.153	0
	−	0	3	13	18	24	25		
	+	6	10	10	14	17	9		
	++	13	16	7	6	1	0		
	+++	26	6	2	0	0	0		
	Positive rate (%)	100	91.43	59.38	52.63	42.86	26.47		
Notch1	Cases, n	45	35	32	38	42	34	47.897	0
	−	4	17	14	18	23	16		
	+	16	6	6	14	13	13		
	++	15	7	10	5	6	5		
	+++	10	5	2	1	0	0		
	Positive rate (%)	91.11	51.43	56.25	52.63	45.23	52.94		
hTERC	Cases, n	25	12	12	12	9	30	81.785	0
	−	0	2	8	12	9	30		
	+	25	10	4	0	0	0		
	Positive rate (%)	100	83.33	33.33	0	0	0		

### 3.2 The correlation between p16^INK4a^ gene and the occurrence and development of cervical cancer is most significant

**Figure 2:**
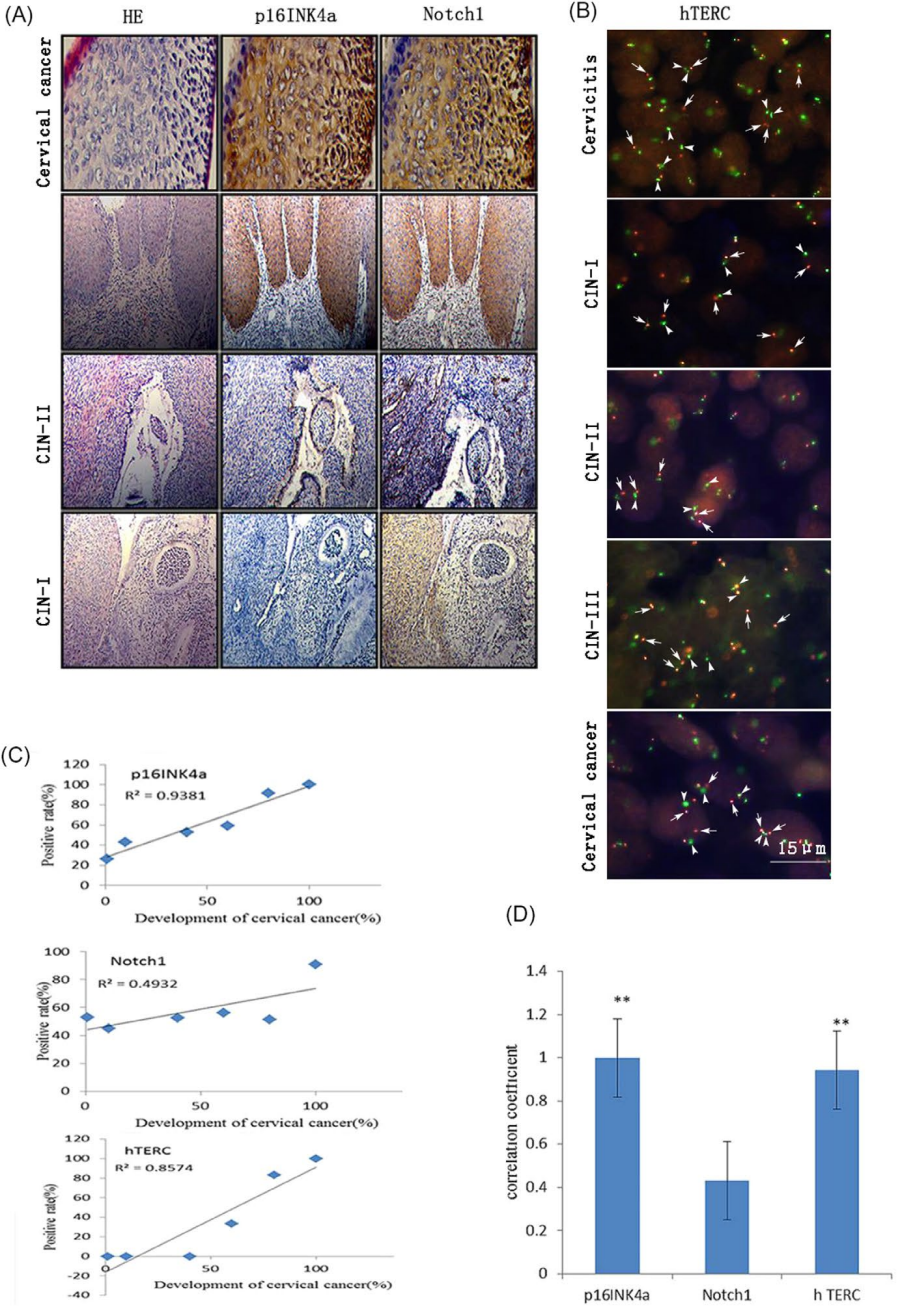
p16INK4a, Notch1, and hTERC genes are associated with the occurrence and development of cervical cancer. A, The expression of p16INK4a and Notch1 genes in the lesion tissues with the occurrence and development of cervical cancer. Immunohistochemical SP method was used. Pale brown was the expression of the corresponding protein, blue was the background of hematoxylin staining, and HE staining was used to locate the lesion. B, The copies of hTERC gene with the occurrence and development of cervical cancer in diseased tissue. Fluorescence in situ hybridization with GLP TERC/CSP3 probe, the green signal for the centromere (arrow heads), the red signal for hTERC gene (arrows). C, The correlation between p16INK4a, Notch1, and hTERC genes with the occurrence and development of cervical cancer was fitted. The occurrence and development of cervical cancer were quantified (cervical cancer—100%, CIN‐III—80%, CIN‐II—50%, CIN‐I—20%, uterine leiomyoma—10%, chronic cervicitis—5%), and the positive expression rates of the three genes in cervical lesions were fitted. D, The correlation coefficients of p16INK4a, Notch1, and hTERC genes with the occurrence and development of cervical cancer were compared. The correlation coefficient was obtained by Spearman rank correlation analysis

The expression of p16^INK4a^, Notch1, and hTERC genes has a positive correlation with the occurrence and development of cervical cancer. In immunohistochemistry, p16^INK4a^ and Notch1 genes were significantly increased in the number and degree of staining cells in tissues (Figure 2A). In fluorescence in situ hybridization (FISH), hTERC gene showed an increase in the number of cells and copies of genes increase progressively (Figure 2B). In Figure 2A, we can find that p16^INK4a^ has high specificity and sensitivity in the occurrence and development of cervical cancer. Compared with HE staining, its expression occurs only at the lesion site, and its expression level is distinct in different stages of cervical cancer development. The expression of Notch1 gene is not regular. There is no differentiation in the expression of the Notch1 gene before cancer, and it is also expressed in non‐lesion sites. According to the positive expression rate of Table 2 in various cervical lesions, it can also be seen that the sensitivity of its expression before cancer is low, and it has little significance for early diagnosis of cervical cancer. According to Table 2 and Figure 2B, the hTERC gene has a certain sensitivity for monitoring the occurrence and development of cervical cancer. But after CIN‐III, it showed obvious amplification and hysteresis, which has great clinical significance in assisting the diagnosis of cervical cancer, and there was no significant difference in the early diagnosis and screening of cervical cancer.

Similarly, the occurrence and development of cervical cancer were quantified in this study (cervical cancer—100%, CIN‐III—80%, CIN–II—50%, CIN–I—20%, leiomyoma—10%, chronic cervicitis—5%). Spearman rank correlation analysis was performed according to the positive expression rates of three genes in Table 2. The results showed that p16^INK4a^ and hTERC genes were significantly correlated with the occurrence and development of cervical cancer, while Notch1 gene had no significant correlation (Figure 2C,D).

In conclusion, the p16^INK4a^ gene has the most significant correlation with the occurrence and development of cervical cancer, which can be considered as a biomarker for early diagnosis and screening of cervical cancer.

### 3.3 The expression of p16^INK4a^ and Notch1 genes in HPV16‐positive cervical cancer is associated with HPV infection

**Figure 3:**
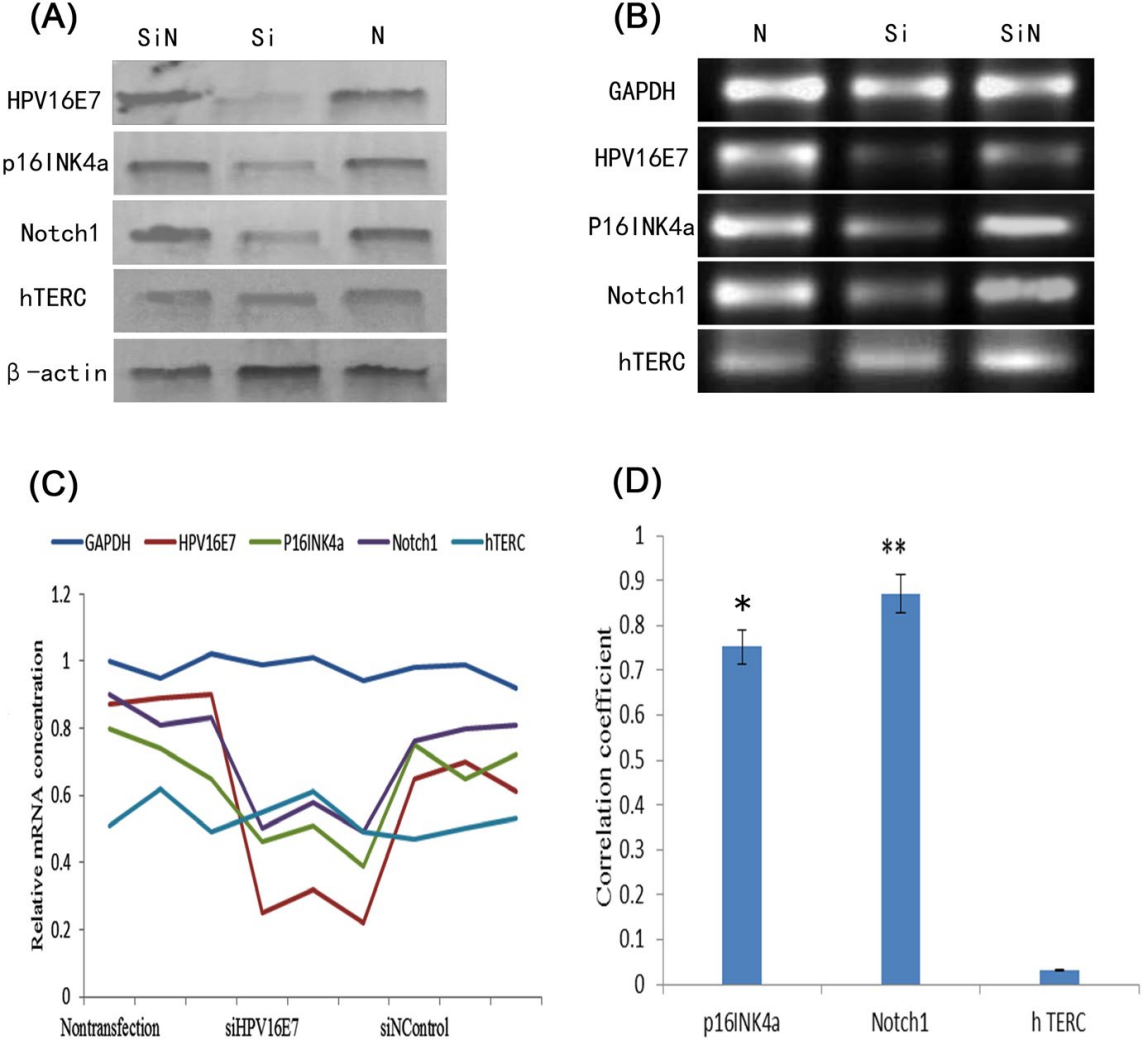
Correlation of p16INK4a, Notch1, and hTERC genes with HPV16 infection. A, The expression of specific proteins after HPV16 E6/E7 SiRNA interfered with HPV16‐positive SiHa cells. Western blot was used to detect the total protein extracted from three groups of SiHa cells after interference, with beta‐actin as the reference. Three groups of cells included nontransfection group, siNControl group: transfection negative control disordered siRNA sequence, siHPV16 E6/E7 group: transfection specific siRNA sequence; B, the expression of specific gene after HPV16 E6/E7 SiRNA interfered with HPV16‐positive SiHa cells. The specific gene was amplified by RT‐PCR, the mRNA was derived from the disturbed SiHa cells, and the PCR product was detected by 2% agarose gel electrophoresis; C, changes in specific gene mRNA levels. The total RNA of SiHa cells was extracted, and the relative mRNA levels of each specific gene were obtained by fluorescence quantitative PCR with reference to GAPDH. D, Comparison of the correlation coefficients between the p16INK4a, Notch1, and hTERC genes and the HPV16 E6/E7 gene. Correlation coefficients were obtained by Spearman rank correlation analysis with respect to the relative mRNA levels of the p16INK4a, Notch1, hTERC, and HPV16 E6/E7 genes

Interference with HPV16 E6/E7 siRNA on HPV16‐positive SiHa cells, as shown in Figure 3A,B, the expression of the HPV16 E6/E7 gene in SiHa cells was inhibited, and the expression levels of HPV16 E6/E7 protein and HPV16 E6/E7 mRNA in the siHPV16 E6/E7 group were significantly lower than those in the nontransfection and siNControl groups. HPV16 E6/E7 siRNA interference plays a significant role. The expression level of the housekeeping gene (GAPDH gene) had no effect before and after interference, indicating that there was no effect on the normal growth of cells before and after interference. The expression levels of Notch1 gene and p16^INK4a^ gene in the interference group also decreased, indicating that RNA interference inhibited the expression of HPV16 E6/E7, which had a certain effect on the Notch1 gene and p16^INK4a^ gene in SiHa cells. Therefore, HPV16 E6/E7 gene was integrated into the genome after HPV infection, and its expression promoted the expression of Notch1 gene and p16^INK4a^ gene. The Notch1 gene and p16^INK4a^ gene were closely related to HPV16 E6/E7 gene. There was no significant difference in the expression of hTERC gene before and after interference, indicating that hTERC gene expression was not associated with HPV16 E6/E7 gene.

As shown in Figure 3C, the relative mRNA levels of p16^INK4a^, Notch1, and hTERC genes were analyzed. The results showed that the mRNA changes of Notch1 gene and p16^INK4a^ gene were very similar to HPV16 E6/E7 gene and had significant correlation (Figure 3D), while hTERC gene was no significant change in mRNA levels.

## 4 DISCUSSION

In the occurrence and development of cervical cancer, HPV virus is the main factor that induces cervical cancer. Most HPV infections and their clinical manifestations will heal after 1‐2 years, but there will still be a small number of infected people with persistent HPV infection, cervical epithelial lesions, and even cervical malignancies.Patients with cervical disease have no significant symptoms in the early stages of onset and are easily ignored. Failure to treat them in time will increase the risk and difficulty of treatment. Therefore, from the perspective of screening and early diagnosis of cervical lesions, there is an urgent need for other reliable indicators or means to assist in the inspection. Make screening more accurate, reliable, and predictable. Atypical squamous cells (ASC) are an indeterminate cytological diagnosis method in the cervical TBS diagnostic system. The degree of cell abnormality is insufficient in quality and quantity to diagnose squamous intraepithelial lesions. But it is more obvious than reactive cells. ASU includes lesions of varying degrees from inflammation to neoplasia in cervical epithelial cells at all levels. About 20% of ASCs were confirmed as high‐grade squamous intraepithelial lesion (HSIL) or even cervical cancer by biopsy. The diagnosis is called "gray area" of gynecological cytological diagnosis.^20^ Screening for the ASC gray area facilitates the treatment of CIN. Therefore, not only combined HPV testing can be used as a method to diagnose the disease,^21^ but also be combined with other biological markers that are closely related to early screening for cervical cancer to improve the sensitivity and specificity of early diagnosis of cervical cancer. Most of the current studies believe that the expression rates of p16^INK4a^, Notch1, and hTERC genes in cervical precancerous lesions and cervical cancer are significantly better than other markers. It is a potential marker for monitoring the risk of cervical precancerous lesions and cervical cancer progression. This study examined the expression of p16^INK4a^, Notch1, and hTERC genes in cervical cancer and precancerous lesions. Expression changes and correlations in the development of cervical cancer and its precancerous lesions were analyzed. It was confirmed that the p16^INK4a^, Notch1, and hTERC genes are closely related to HPV‐infected cervical precancerous lesions and cervical cancer. The value of p16^INK4a^, Notch1, and hTERC genes as molecular biological indicators for the development of cervical cancer and its precancerous lesions was compared.

The p16^INK4a^ gene is a tumor suppressor gene involved in cell cycle regulation and participates in the regulation of the cell cycle from G1 to S phase. Inactivation of the p16^INK4a^ gene is widespread in a variety of human tumor cells. The mechanism of inactivation mainly includes deletion, mutation, and abnormal methylation.

Studies have shown that p16^INK4a^ gene expression is frequently absent in most malignant tumors. However, deletion and mutation of p16^INK4a^ gene are rare in cervical cancer and precancerous lesions, mainly expressed as overexpression. Studies have shown that high expression of E6/E7 in HPV16 can trigger tumorigenic signaling and induce epigenetic genetic alterations, especially the CDKN2A (p16^INK4a^/ARF) locus, resulting in overexpression of a large number of p16^INK4a^ proteins. The p16^INK4a^ protein is closely associated with high‐risk HPV infection in cervical precancerous lesions and cervical cancer tissues.^22^ High‐risk HPV infection leads to proliferation of cervical cancer cells and overexpression of p16^INK4a^, and overexpression of p16^INK4a^ can be detected by immunohistochemistry. Currently, as one of the markers of HPV‐induced transformation,^23^, ^24^ high levels of p16^INK4a^ expression can also maintain the growth of tumor HPV‐switched cells.^24^ Studies show that the positive expression of p16^INK4a^ in different cervical lesions in Chinese women is as follows: cervical cancer, CIN‐III, CIN‐II, CIN‐I, and the positive expression rates in the control group are 99.37%, 99.46%, 98.83%, 61.53%, and 19.21%. Cervical cancer, CIN‐III, CIN‐II, no significant difference between the three groups, the positive expression rate was over 97.9%, p16^INK4a^ can effectively identify CIN‐II+ lesions. However, in this study, the positive expression rates of p16^INK4a^ gene in cervical cancer, CIN‐III, CIN‐II, CIN‐I, uterine leiomyoma, and chronic cervicitis were 100.00% (45/45) and 91.43% (32/35), 59.38% (19/32), 52.63% (20/38), 42.86% (18/42), and 26.47% (9/34), which are significantly related to the occurrence and development of cervical cancer in Mongolians (*R* = 0.913, *P* < .01), and there was a significant correlation with HPV16 infection (*r* = 0.753, *P* < .05), indicating that the p16^INK4a^ gene shows high specificity and sensitivity. However, the expression level of p16^INK4a^ gene in Mongolian patients with cervical lesions is slightly different from previous reports. There is no significant difference between cervical cancer and CIN‐III groups, and CIN‐II group is significantly lower than cervical cancer and CIN‐III groups. p16^INK4a^ can effectively identify CIN‐III + lesions rather than CIN‐II + lesions. Similarly, the expression level of the p16^INK4a^ gene in HPV16‐infected cervical cancer and precancerous lesions was prominent in this study. The positive expression rates of p16^INK4a^ gene in cervical cancer, CIN‐III, CIN‐II, CIN‐I, uterine leiomyoma, and chronic cervicitis were 100.00% (45/45), 91.43% (32/35), 59.38% (19/32), 52.63% (20/38), 42.86% (18/42), and 26.47% (9/34), respectively. The correlation with the occurrence and development of cervical cancer was extremely significant (*r* = 0.913, *P* < .01), and the correlation with HPV16 infection was significant (*r* = 0.753, *P* < .05), showing high specificity and sensitivity.

Laura et al believe that the carcinogenesis of normal cervical epithelium may be related to the increased expression of Notch1 protein, leading to the development of cervical cancer.^11^ Notch1 was detected in research related to cervical cancer more than a decade ago, suggesting that it may play a role in cervical cancer. Notch1 can cooperate with HPV E6 and E7 oncogenes to promote malignant transformation of cervical squamous epithelial cells. Conversely, other studies have shown that Notch1 has a tumor suppressive effect in cervical cancer. Its dual effects may be related to Notch1 concentration and cervical lesions at different stages. In this study, the expression of the Notch1 gene was expressed in cervical cancer, CIN‐III, CIN‐II, CIN‐I, uterine leiomyoma, and chronic cervicitis. The positive expression rate of Notch1 protein was 91.11% (41/45), 51.43% (18/35), 56.25% (18/32), 52.63% (20/38), 45.23% (19/42), and 52.94% (18/34). However, we found that the expression of the Notch1 gene is not very regular. There is no discrimination in the expression of precancerous cells, and it is also expressed in non‐lesional sites. It is only associated with HPV16 infection, and the sensitivity of precancerous expression is low. It has little significance in the early diagnosis of cervical cancer.

The National Institutes of Health research in recent years on cervical cancer shows that the majority of cervical epithelial cell carcinogenesis is accompanied by an increase in 3Q copies. The human telomerase RNA component (hTERC gene, located at 3q26.3) may be the most important gene involved.^19^ Abnormal expansion of the hTERC gene is thought to be an early event in the development of cervical cancer. The hTERC gene amplification can prevent apoptosis, which leads to tumor production. In this study, we also proved that the hTERC gene is closely related to Mongolian patients with cervical cancer. However, there was no significant association with HPV16 infection. The hTERC gene has a certain sensitivity for monitoring the occurrence and development of Mongolian patients with cervical cancer. But after CIN‐III, it showed obvious amplification and hysteresis, which has great clinical significance in assisting the diagnosis of Mongolian patients with cervical cancer, and there was no significant difference in the early diagnosis and screening of Mongolian patients with cervical cancer.

High‐risk papillomavirus (HPV) infection and persistent expression of E6/E7 oncogene are highly associated with cervical cancer. Activated Notch‐1 signa**l**ing pathway can cooperate with E6/E7 to induce tumorigenesis.^25^ However, there was no additional change in cell DNA. HPV E6 protein and E7 protein still could not completely transform into the normal cervical cell. Mainly reflected in three points: first, the peak age of cervical cancer is 40 years old, far behind the peak age of HPV infection (about 25 years old)^26^; second, although HPV infection is more common, only a few women eventually develop cervical cancer after HPV infection; third, different individuals in the same environment have different HPV infection susceptibility and susceptibility to cervical cancer. Therefore, our research on the carcinogenic mechanism of HPV infection is very necessary. The disorder of Notch1 signaling activity may be one of the factors that cause additional changes in cervical cell DNA, leading to complete cell transformation.^27^, ^28^, ^29^ It is not clear how the p16^INK4a^ and hTERC genes interact with HPV16 E6/E7 to cause cervical cancer.

This study interfered with HPV16‐positive SiHa cells by HPV16 E6/E7 siRNA. The results showed that the expression of HPV16 E6/E7 promoted the expression of Notch1 gene and p16^INK4a^ gene. Notch1 gene and p16^INK4a^ gene were closely related to HPV16 E6/E7 gene, while hTERC gene expression was not associated with HPV16 E6/E7 gene.

## AUTHOR CONTRIBUTIONS

All the authors have accepted responsibility for the entire content of this submitted manuscript and approved the submission.

## ETHICAL APPROVAL

This study was approved by the Institutional Review Board of the affiliated hospital of Inner Mongolia University for the Nationalities and complied with the Declaration of Helsinki. The written informed consent was obtained from each participant.
