# Supplementary material for: Relevance research between the expression of p16INK4a, Notch1, and hTERC genes: The development of HPV16‐positive cervical cancer
Source: J Clin Lab Anal. 2020 Jan 24;34(5):e23207. doi: 10.1002/jcla.23207 (PMC7246350; doi:10.1002/jcla.23207)
Supplement: Supplementary file 1 [file JCLA-34-e23207-s001.doc]

TABLE S1 Overview the NO. and age of the patients

| Degree of lesion | NO. of patients | Age range | Average age |
| --- | --- | --- | --- |
| Cervical cancer | 45 | 28-71 | 50.88 |
| CIN-Ⅲ | 35 | 22-58 | 42.44 |
| CIN-Ⅱ | 32 | 20-47 | 40.29 |
| CIN-Ⅰ | 38 | 23-63 | 40.35 |
| Uterine leiomyoma | 42 | 25-52 | 43.57 |
| Chronic cervicitis | 34 | 26-63 | 44.81 |

Note: All samples were positive for HPV16 infection.
